# Spread of Vector-borne Diseases and Neglect of Leishmaniasis, Europe

**DOI:** 10.3201/eid1407.071589

**Published:** 2008-07

**Authors:** Jean-Claude Dujardin, Lenea Campino, Carmen Cañavate, Jean-Pierre Dedet, Luigi Gradoni, Ketty Soteriadou, Apostolos Mazeris, Yusuf Ozbel, Marleen Boelaert

**Affiliations:** * Instituut voor Tropische Geneeskunde, Antwerp, Belgium; † Instituto de Higiene e Medicina Tropical, Lisbon, Portugal; ‡ Instituto de Salud Carlos III, Madrid, Spain; §Université Montpellier 1, Montpellier, France; ¶ Istituto Superiore di Sanità, Rome, Italy; # Hellenic Pasteur Institute, Athens, Greece; ** National Reference Laboratory for Animal Health, Nicosia, Cyprus; †† Ege University Medical School, Bornova, Izmir, Turkey

**Keywords:** leishmaniasis, Europe, surveillance, neglected diseases, malaria, perspective

## Abstract

The risk for reintroduction of some exotic vector-borne diseases in Europe has become a hot topic, while the reality of others is neglected at the public health policy level. Leishmaniasis is endemic in all southern countries of Europe, with ≈700 autochthonous human cases reported each year (3,950 if Turkey is included). Asymptomatic cases have been estimated at 30–100/1 symptomatic case, and leishmaniasis has up to 25% seroprevalence in domestic dogs. Even though leishmaniasis is essentially associated with *Leishmania infantum* and visceral leishmaniasis, new species, such as *L. donovani* and *L. tropica*, might colonize European sand fly vectors. Drug-resistant *L. infantum* strains might be exported outside Europe through dogs. Despite this possibility, no coordinated surveillance of the disease exists at the European level. In this review of leishmaniasis importance in Europe, we would like to bridge the gap between research and surveillance and control.

## Abstract 2

Exotic vector-borne diseases are gaining attention at the expense of leishmaniasis.

In August through September of 2007, a chikungunya outbreak occurred in the province of Ravenna, Italy (*1*). The risk for reintroduction of vector-borne diseases in Europe as a consequence of global warming was highlighted, although long-distance tourism, travel, and trade could also play major roles in the transcontinental transport of microorganisms (*2*). The European Centre for Disease Control is currently assessing the magnitude and importance of vector-borne diseases in Europe, focusing on Lyme disease, tick-borne encephalitis, leptospirosis, malaria, plague, tularemia, viral hemorrhagic fevers, hantavirus, and West Nile fever. Concern about the impact of global warming and the spread of arthropod-borne diseases and other infectious agents in Europe is justifiable. However, existing autochthonous vector-borne infections should not be forgotten or ignored, which may be the case, as illustrated here for leishmaniasis.

## Leishmaniasis in Europe

Leishmaniasis is a major vector-borne disease, which is endemic in 88 countries and is the only tropical vector-borne disease that has been endemic to southern Europe for decades. In southern Europe, most of the reported cases are due to zoonotic visceral leishmaniasis (VL), which is the most dangerous form and is lethal when untreated. Cutaneous leishmaniasis (CL), which is more benign than VL, is also present. Incidence of leishmaniasis in humans is relatively low, ranging from 0.02/100,000 to 0.49/100,000 (8.53/100,000 including Turkey). We estimate that this corresponds to a total of ≈700 reported new cases per year for southern European countries (3,950 if Turkey is included; Table and Figure). However, autochthonous leishmaniasis appears not to be limited to the Mediterranean region anymore. It has SPREAD northward, as shown by the recent reports of indigenous VL cases in northern Italy and southern Germany (*8**,**9*).

**Table:** Leishmaniasis situation in 7 disease-endemic countries of Europe (including Turkey)*

Country	Human leishmaniasis				Canine leishmaniasis
	Notification status	Current information from reference centers (2000–2006)	VL + CL incidence x 100,000†	Imported cases (VL + CL)	
Portugal‡	Compulsory for VL	≈22 VL cases/y recorded at IHMT	0.07–0.17	≈2 cases/y recorded at IHMT	Average 20% seroprevalence in disease-endemic areas (*3*)
Spain§	Compulsory in 12/17 autonomous communities; 20%–45% underreporting for VL, ≈100% for CL (*4*)	≈100 VL cases/y recorded by National Epidemiologic Surveillance Network, RENAVE	0.18–0.29	≈5 cases/y recorded at ISCIII	Average 8.5% seroprevalence (*5*)
France¶	Not compulsory, but spontaneous reports at UMON	≈24 VL + CL cases/y reported at UMON	0.02–0.19	≈65 cases/y recorded at UMON	Seroprevalence in disease-endemic areas of southern France 4%–20%#
Italy**	Compulsory for both VL and CL, but CL underreported	≈200 VL cases/y recorded at ISS; ≈300 CL cases/y estimated by ISS	0.15–0.38	≈8 cases/y recorded at ISS	Average 15% seroprevalence in peninsular Italy; average 2% seroprevalence in continental Italy (*6*)
Greece††	Compulsory for both VL and CL, but underreported	≈21 VL cases/y notified	0.06–0.49	Unknown	Average seroprevalence 25% in disease-endemic areas (*7*)
Cyprus‡‡	Compulsory for both VL and CL, but underreported	5 VL + CL cases recorded in 2006	0.25–0.47	Unknown	Average seroprevalence 20% in disease-endemic areas
Turkey§§	Compulsory for both VL and CL	≈37 VL cases/y and ≈2,300 CL cases/y notified	1.6–8.53	Unknown	Average 15.7% seroprevalence

*Authors’ institutions are national reference laboratories for leishmaniasis diagnosis and surveillance and rely on consolidated countrywide networks of collaborating clinical health centers. Diagnosis records are cross-checked with case notifications to provide more realistic figures and estimates. VL, visceral leishmaniasis; CL, cutaneous Leishmaniasis; WHO, World Health Organization. †WHO-EURO, WHO Europe, 1996–2005; http://data.euro.who.int/CISID.  ‡Instituto de Higiene e Medicina Tropical (IHMT), Lisbon, Portugal. §Instituto de Salud Carlos III (ISCIII), Madrid, Spain. ¶Université de Montpellier (UMON), data from Centre National de Référence des Leishmania, Montpellier, France. #Source: retrospective canine leishmaniasis database, Centre National de Référence des Leishmania. **Istituto Superiore di Sanità (ISS), Rome, Italy. ††Hellenic Pasteur Institute (HPI), Athens, Greece. ‡‡National Reference Laboratory for Animal Health (VS), Nicosia, Cyprus. §§Ege University (EUMS-DP), Izmir, Turkey.

**Figure:**
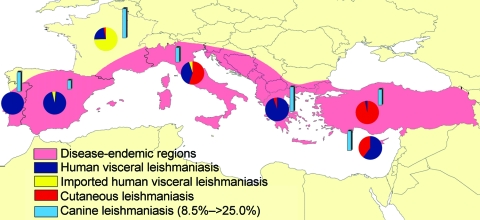
Leishmaniasis in southern Europe. Distribution of the endemic disease; relative proportion of autochthonous (visceral, cutaneous) and imported human cases and seroprevalence in dogs (from data reported in Table).

However, these numbers are misleading for several reasons. First, data from patients infected in southern Europe, but diagnosed elsewhere, are not taken into consideration. For instance, a leishmaniasis reference center established on a voluntary basis in Germany identified within 2 years 70 cases of leishmaniasis. Of the 27 VL case-patients, most (*17*) had been infected within European Union boundaries: Spain, Portugal, Greece, or France (*10*). Five cases were in children. Similarly, a retrospective study in the Hospital for Tropical Diseases in London showed that most of the imported VL case-patients in the United Kingdom were adult men touring the Mediterranean (*11*). Second, in the absence of public health surveillance at the European level, underreporting is common (see the Leishmaniasis and the Globalization of Neglect section). Third, asymptomatic infections may be common in some regions: for 1 clinical case of VL, there may be 30–100 subclinical infections (*12*). This underreporting can have major consequences for blood banks: blood from donors living in areas of endemicity in southern France and Greece had 3.4% and 15% seropositivity, respectively (*13*,*14*). In addition, 22.1% of blood donors in a highly disease-endemic area from Spain were PCR positive for leishmaniasis (*15*). Furthermore, asymptomatic infections may progress to severe clinical forms in immunocompromised persons, for example, in AIDS patients (*16*). Fourth, the etiologic agent of southern European VL, *Leishmania infantum*, is also infecting dogs (with a seroprevalence of up to 34% in areas of Spain where the disease is highly endemic) (Table). Dogs with leishmaniasis infections are generally very sick, causing a major problem in southern Europe (e.g., ≈5,000 clinical cases occur each year in France) (Table). However, sick as well as asymptomatic dogs also represent a risk for humans, as they constitute the major reservoir of the parasite on which sand fly vectors may feed and transmit the infection.

## Import–Export Balance of European Leishmaniasis

In addition to the reality of autochthonous leishmaniasis in Europe, the risk for introduction of new species through travelers or immigrants from countries where non-European species are endemic should also be considered. However, the probability that these species could enter in a transmission cycle is relatively low. The probability depends on contact between infected persons and sand flies, the capacity of the infected person to act as reservoir, and the susceptibility of European sand flies to the different *Leishmania* species. For most species, humans are generally a transmission dead-end. However, for 2 species, the risk might theoretically be higher: *L. tropica*, which is causing CL in Africa, the Middle East, and Southwest Asia, and *L. donovani*, the etiologic agent of VL in East Africa and the Indian subcontinent. These 2 species are indeed associated with an anthroponotic transmission cycle. On one hand, *L. donovani,* which is transmitted by a different species of sand fly outside Europe, might be hosted by most European sand flies, except *Phlebotomus papatasi* and *P. sergenti* (*17*). On the other hand, *L. tropica*, which has more stringent requirements in terms of vector, would need *P. sergenti*, which was reported in several places in southern Europe, from Portugal (*18*) to Cyprus (*19*). *L. tropica* was indeed encountered in Greece (*20*), and according to a very recent report, the first autochthonous cases of *L. donovani* in Europe have been detected in Cyprus (*21*). The clinical phenotype associated with both species needs also to be considered for an exhaustive risk evaluation. *L. tropica* causes lesions that are generally more difficult to treat with antimonial drugs (*22*), whereas *L. donovani* is considered to be more aggressive than *L. infantum* and often does not respond to treatment with first-line drugs (*23*).

In addition to being concerned about importation and spread of exotic *Leishmania* species in Europe, exportation should also be considered. The best known historical example of the spread of leishmaniasis is the migration of *L. infantum* from Europe to Latin America, where it colonized in *Lutzomyia longipalpis* and is now causing a serious public health problem (>3,500 cases of VL per year in Brazil) (*24*). This spread is thought to have been caused by conquistadores’ dogs (*25*). Another and current example concerns the *L. major/L. infantum* hybrids recently described in HIV-positive VL patients from Portugal (*26*). Indeed, these hybrids were shown to be able to develop in *P. papatasi* (*27*), a vector that is widespread in Europe, Africa, and Asia. Considering the reservoir role of HIV–co-infected patients and the peridomestic and anthropophilic nature of *P. papatasi*, these hybrid strains might circulate by using this sand fly vector, thereby increasing the risk of their spreading into new foci throughout the broad range of *P. papatasi* distribution (*27*). Finally, the way Europe deals with its leishmaniasis public and animal health problem can still have major consequences for the rest of the world. Miltefosine, one of the few available antileishmania drugs, has been recently launched in the market for canine leishmaniasis treatment in Portugal, Spain, Italy, Greece, and Cyprus. Because dogs are never cured parasitologically and given the long half-life of the drug, the lack of European policy might contribute to the emergence of parasites resistant to miltefosine. This resistance could be a problem for European human patients, as miltefosine is being used on a compassionate basis in several European AIDS co-infected patients unresponsive to amphotericin B or pentavalent antimonials (*28*,*29*). Furthermore, if dogs infected with miltefosine-resistant strains were to migrate to Latin America, where several countries have registered the drug for human use (currently Colombia, Guatemala, Argentina, Venezuela, Paraguay, Ecuador, and Honduras; *30*), the impact might be greater.

## Leishmaniasis and the Globalization of Neglect

Twelve million persons have leishmaniasis, and 500,000 new cases of VL occur each year. More than 50,000 die of this disease each year. The disease is spreading because of several risk factors, climate being only one. Humanmade changes to the environment and population movements (for economic or political reasons) may lead to alterations in the range and densities of the vectors and reservoirs, increasing human exposure to infected sand flies. Urbanization of leishmaniasis becomes more common and in conjunction with the ruralization of HIV/AIDS, it contributes to increase the problem of co-infections in contexts where access to highly active antiretroviral therapy is not the same as in industrialized countries. *Leishmania* spp. have already become resistant to antimonial drugs (the first-line drug in many developing countries) in some regions and may soon become resistant to miltefosine (*23*). Despite this increasing resistance, leishmaniasis is one of the most neglected diseases in developing countries, along with others like sleeping sickness or Chagas disease. Leishmaniasis is a disease for which we lack effective, affordable, and easy to use drugs, and the pharmaceutical industry has had few incentives to engage in their development. In addition, leishmaniasis surveillance and control are also neglected. One of the main reasons for this neglect is that in developing countries, leishmaniasis is a disease of the poor. Risk for infection and clinical development are mediated by poverty, while leishmaniasis diagnosis and treatment are expensive and may lead to further impoverishment and reinforcement of the vicious cycle of disease and poverty (*31*).

In Europe, physicians are sometimes ill-informed on the diagnosis and treatment of leishmaniasis. In France, a telephone advice line was created in 2006 by the National Reference Centre of Leishmania to help physicians in their therapeutic diagnosis. A study in Germany, a non–disease-endemic country, showed that the median time between symptom onset and correct diagnosis was 85 and 61 days in case-patients of VL and CL, respectively (*32*). This value was lower in a leishmaniasis-endemic area, such as southern Italy (35 days, [*33*]). VL, which was initially a pediatric disease in Europe (hence the name of *L. infantum*), only began to gain attention when the co-infection of HIV/AIDS was documented. Between the late 1980s and early 2001, >1,900 cases were reported in southwestern Europe (*16*). Even though it was reported that both pathogens could be transmitted through sharing of needles among intravenous drug users (*34*), in many cases of co-infection, the parasite was already present at the time of HIV infection, which indicates that HIV infection would have an unmasking effect on the true endemicity of *Leishmania* infection. In other words, the wave of *Leishmania*/HIV co-infection showed that *L. infantum* could behave as an opportunistic parasite, with many asymptomatic carriers (*12*), and with the clinical syndromes being only the tip of the iceberg. Because of the highly active antiretroviral therapy, cases of co-infection generally decreased in the region, with the exception of Portugal (*35*).

Notification of VL varies according to the country. It does not belong to the list of 30 notifiable diseases in France. However, notification is compulsory in Greece, Italy, and Portugal, though only obligatory in 12 of 17 autonomous communities of Spain. Underreporting is common. In Portugal, for instance, 76 cases of autochthonous VL were officially reported at the country level from 2000 through 2005. During the same period, 127 cases (+67%) were observed in the Institute of Tropical Medicine of Lisboa (Table). In the case of autochthonous cutaneous leishmaniasis, consolidated data are lacking, but this clinical form is definitely underreported because of its benign nature and the fact that it usually does not require hospitalization. Nonetheless, leishmaniasis is not a disease placed under public health surveillance at the European level. It does not even belong to the package of rare diseases considered as a priority in the Public Health Programme 2003–2008. (Rare diseases, including those of genetic origin, are life-threatening or chronically debilitating diseases that are of such low prevalence [<5/10,000 persons] that special combined efforts are needed to address them so as to prevent significant illness or perinatal or early deaths or a considerable reduction in a person’s quality of life or socioeconomic potential.) At the regional level, the only dedicated network of surveillance was the one launched by the World Health Organization and the Joint United Nations Programme on HIV/AIDS in 1993 for the surveillance of *Leishmania*/HIV co-infections, which essentially involved European countries as well as some developing countries.

The low-profile perception seen for human leishmaniasis differs dramatically from the veterinary world’s perception. The high incidence of canine leishmaniasis in southern Europe makes *Leishmania* one of the main dog killers in the region, and private veterinarians are well aware of it. Dogs are treated individually to protect from sand fly bites, and those diagnosed as infected are considered extremely difficult to treat. Specific web sites are available for owners of infected dogs to discuss and compare treatment regimens and pose questions to veterinarians. Several pharmaceutical companies are investing in research and development of vaccines, drugs, and topical insecticides for specific cure and prevention of canine leishmaniasis. This high-profile perception, however, drops when dogs must be treated as the reservoir of human leishmaniasis. For instance, the issue of notification is treated differently in various leishmaniasis–endemic countries, but even where notification is compulsory (i.e., Italy and Spain), it is not a common practice. In Italy, the network Leishmap is currently monitoring the spread of canine leishmaniasis and vectors in northern Italy. Leishmap is a scientific network, supported by a private company (*36*). Furthermore, private interests are sometimes at odds with public health goals. Drugs for leishmaniasis are not regulated in the veterinary market, and medications intended for use in humans, such as Ambisome, are used in domestic pets, with the potential risk that they might be a source for the emergence and spreading of resistant strains.

## Countering the Neglect

Since 2001, several research consortia gathered scientists from Euro-Mediterranean countries (www.leishrisk.net). These consortia and other research groups generated knowledge, tools, and education packages and led to a solid European research network dedicated to the study of leishmaniasis. Bridging research with surveillance and control is an issue of dialogue and advocacy. On one hand, health professionals need to be in close contact with scientists to help translate basic research into relevant and applicable tools. For instance, sequencing the whole genome of *Leishmania* represented a technologic challenge, but the next challenge is to exploit this sequencing for the benefit of the patient (www.leishrisk.net). On the other hand, scientists must market their results to influence health policy. Changes in health policy are being made; during manuscript revision, we were informed of the selection of leishmaniasis among the priority zoonoses addressed by the Episouth network (www.leishrisk.net).

Deciding health policy is a complex social, economic, and political interrelationship that is much broader than leishmaniasis alone (or even infectious diseases generally). However, if Europe justifiably wants to invest more in surveillance of vector-borne diseases, the time has come to recognize its real impact on both animal and human health and include leishmaniasis as one of these diseases.
